# A novel corrected anion gap links metabolic acid attack to peptic ulcer disease

**DOI:** 10.3389/fnut.2026.1758803

**Published:** 2026-01-14

**Authors:** Xian-Ping Lin, Song-Xing Lin

**Affiliations:** 1 School of Nursing, Putian University, Putian, Fujian, China; 2 Health Management Center, Affiliated Hospital of Putian University, Putian, Fujian, China

**Keywords:** acid-base balance, anion gap, corrected anion gap, metabolic acid load, metabolic non-volatile acid, peptic ulcer disease, unmeasured anions

## Abstract

**Background:**

Physiological evidence indicates that systemic acid-base balance may be associated with gastric acid secretion, which is a key factor in peptic ulcers disease (PUD). However, the epidemiological relationship of this association with PUD risk remains unclear.

**Aim:**

This study investigates a novel corrected anion gap (AG_corr_, reflecting metabolic acid load) and bicarbonate (HCO₃^−^, reflecting alkaline reserve) in relation to PUD.

**Methods:**

This retrospective case-control study analyzed endoscopy-confirmed 64 PUD patients and 541 non-PUD controls with normal hepatic and renal function from a Fujian tertiary hospital (Jan 2023–Jun 2025). The anion gap (AG) was corrected to fully-corrected AG_corr_ and albumin-corrected AG (ACAG). Four models (A: AG_corr_; B: HCO₃^−^; C: AG; D: ACAG) were adjusted for sex, age, and Na to study the relationships between AG_corr_, ACAG, AG, HCO_3_, and PUD. Post-hoc power and sensitivity analysis were conducted.

**Results:**

Model A showed that AG_corr_ was significantly positively associated with PUD risk (OR = 1.167, 95% CI: 1.050–1.298, *P* = 0.045), with a high statistical power of 89%. Sensitivity analysis with an expanded control group (*n* = 1915) confirmed a significant AG_corr_-PUD association (OR = 1.138, 95% CI: 1.029–1.259, *P* = 0.012), with post hoc power of 0.81. The sensitivity analysis with case-control matching for sex and age showed that AG_corr_ remained significantly associated with PUD (*P* = 0.020). In contrast, Model C and Model D were both significantly correlated (*P* < 0.05) but showed poor fit (H-L *P* < 0.05). However, their statistical power differed, with values of only 0.64 and 0.84, respectively. Model B indicated a negative correlation between PUD and HCO₃^−^ (OR = 0.894, 95% CI: 0.802–0.996, *P* = 0.043), but with low statistical power (65%) and instability in sensitivity analysis.

**Conclusion:**

After adjusting for sex, age, and Na, AG_corr_ remained an independent risk factor for PUD in individuals with normal liver and kidney function. The fully-corrected AG outperformed both ACAG and uncorrected AG. Metabolic non-volatile acid attack may be an important pathogenic mechanism in PUD. HCO₃^−^ may have a potential protective effect against PUD. The findings contribute to improving PUD care but require further validation in diverse groups.

## Introduction

Peptic ulcer disease (PUD) is a common clinical condition. An investigation spanning 1997 to 2005 reported a PUD incidence of 7.5% in the United Kingdom (1). More recent health survey data from 2017 to 2018 indicated that the prevalence of gastric and duodenal ulcers among Chinese adults aged 18–64 was 5.4% (3.9–7.3%) and 2.5% (1.7–3.7%), respectively (2). *Helicobacter pylori* (*H. pylori*), non-steroidal anti-inflammatory drugs (NSAIDs), gastric acid, and pepsin are recognized as the most significant aggressive factors in PUD (3–9). Current understanding of PUD risk focuses predominantly on gastric-localized elements and external triggers, with corresponding therapeutic strategies largely targeting these offenders (10–15). Yet, the role of metabolic acid load, originating from internal metabolic processes, has been largely overlooked. Excessive gastric acid secretion is a recognized invasive factor in PUD pathogenesis. The acid-secreting function of the gastric mucosa may also play a significant role in regulating metabolic acid–base balance. Evidence suggests a potential link between metabolic acid load and gastric hyperacidity in PUD (16, 17). A diet rich in sulfur-containing amino acids and low in fruits and vegetables can adversely affect acid–base balance, potentially leading to subclinical metabolic acidosis (18). Furthermore, normal physiological activities, particularly strenuous exercise, generate acidic metabolites such as lactic acid and ketone bodies.

Metabolic acidosis can be classified into two primary categories: increased load of metabolic acids (predominantly non-volatile acids) and excessive alkali loss. Clinically, the anion gap (AG) serves as an important indicator for assessing metabolic acid load, while bicarbonate (HCO₃^−^) level is a key marker for evaluating alkali reserve. The AG represents the difference between undetermined anions (UA) and undetermined cations (UC) in plasma. When the AG value exceeds the normal range, it suggests a potential increase in non-volatile metabolic acid load, primarily stemming from non-volatile acid drugs, food sources or metabolic disorders, which generate these non-volatile acid products. This pattern is commonly observed in conditions like lactic acidosis and ketoacidosis (19–22). In such cases, because the excess H^+^ ions neutralize HCO₃^−^, the measured HCO₃^−^ level may appear normal or only slightly decreased. Conversely, in specific situations such as renal tubular acidosis or severe diarrhea leading to substantial HCO₃^−^ loss, the metabolic acidosis is characterized primarily by HCO₃^−^ depletion (23). Here, HCO₃^−^ decreases significantly, and chloride (Cl^−^) compensates electroneutrality, resulting in a normal AG. Additionally, acidic substances found in foods like carbonated beverages are easily metabolized and eliminated via respiration, while acetic acid (from vinegar) is readily metabolized in the liver via the tricarboxylic acid (TCA) cycle. Such volatile acids generally do not cause an elevated AG.

The traditional method for calculating AG employs a simplified formula: AG = [Na^+^] – ([Cl^−^] + [HCO₃^−^]) (24), with a normal reference range of 8–16 mmol/L (25). This approach does not account for the influence of substances such as albumin (ALB) and phosphate in plasma, nor does it consider cations like K^+^, Mg^2+^, or Ca^2+^, leading to potential inaccuracies in assessing metabolic acid load. Consequently, researchers often apply AG correction based on specific requirements. A common clinical method is the ALB-corrected AG (ACAG). ACAG may offer improved utility in detecting hyperlactatemia (26). The adjusted AG served as a more sensitive indicator of ionic concentration changes, thus highlighting the importance of this correction (27–30). AG correction plays a significant role in the diagnosis, management, and prognostic evaluation of various diseases (31–33) and has been successfully applied in clinical cases to guide effective treatment (34–36). However, while ACAG is convenient for clinical application, there remains room for enhancing its sensitivity in scenarios demanding high precision—one of the key issues this study aims to address.

Another key indicator of the body’s acid–base balance is HCO₃^−^, which plays a critical role in maintaining this equilibrium. HCO₃^−^ directly measures the body’s alkaline reserve buffering capacity. Its primary mechanisms of action include the following: Firstly, as a major buffer in the blood, HCO₃^−^ forms a buffer pair with carbonic acid (H₂CO₃). When acidic substances increase, H^+^ combines with HCO₃^−^ to form H₂CO₃, which subsequently dissociates into CO₂ and H₂O; the CO₂ is then eliminated via respiration to maintain balance. Conversely, when alkaline substances increase, OH^−^ reacts with H₂CO₃ to generate HCO₃^−^ and H₂O, thereby buffering the alkaline shift (37). In the kidneys, the precise reabsorption and secretion of HCO₃^−^ by renal tubules finely regulate its plasma concentration. Furthermore, abnormalities in intestinal absorption or loss of HCO₃^−^ can also lead to acid–base disturbances (37).

Studies have demonstrated that gastric acid secretion is significantly influenced by systemic acid–base balance, which is closely tied to blood acid load (38–40). This implies that an increased metabolic acid load may exacerbate gastric acid attack. Could PUD pathogenesis therefore be associated with acid load at the systemic level? To address this question, this retrospective case–control study analyzes the relationship between serum AG, HCO₃^−^, and PUD, exploring the role of systemic acid–base imbalance in PUD. To minimize the confounding effects of hepatic and renal dysfunction, the study was conducted in a population with normal liver and kidney function, aiming to observe their independent associations. Additionally, to address the theoretical negative collinearity between AG and HCO₃^−^, a dual-model strategy was employed: an AG model to specifically evaluate the effect of metabolic acid load, and an HCO₃^−^ model to independently assess the role of alkaline reserve status. Moreover, to reduce the estimation error associated with AG for assessing metabolic acid load, we corrected AG to avoid masking any potential effects. Finally, post-hoc power analysis was conducted to quantitatively evaluate the robustness of the statistical findings.

## Methods

### Study design and participants

A retrospective case–control study was conducted using data from individuals who underwent comprehensive biochemical testing and gastroscopy at a tertiary hospital in Fujian Province. Data on biochemical tests and gastroscopy results were retrieved from the hospital’s health examination system for the period from January 14, 2023, to June 30, 2025. Participants were categorized into two groups based on gastroscopic findings: those diagnosed with active peptic ulcer (including gastric or duodenal ulcers with clear active-stage classification A1 or A2) were assigned to the case group, while those with no abnormal findings were assigned to the control group.

### Ethical approval

It was approved by the Putian University Institutional Review Board (Approval No. 2024044). The requirement for informed consent was waived because this was a retrospective analysis of de-identified medical records, posed minimal risk to participants, and the research could not practicably be carried out without the waiver. All data were anonymized prior to analysis, and patient confidentiality was strictly maintained in accordance with national regulations on human subjects research.

### Inclusion and exclusion criteria were as follows

(1) Case group inclusion: Endoscopically diagnosed peptic ulcer (gastric or duodenal ulcer) with documented active stage (A1 or A2).
(2) Control group inclusion: The endoscopic findings comprised solely of a single diagnosis of gastric mucosal status (atrophic or non-atrophic) and did not include any abnormal lesions (such as erosions, acute inflammation, leukoplakia, etc.).
(3) Common exclusion criteria (both groups):

- Incomplete clinical data.
- Endoscopic findings indicating esophageal/gastric varices, hiatal hernia, bleeding, esophagitis, or any malignancy.
- Age < 18 or ≥ 80 years.
- Abnormal liver or kidney function, defined as alanine aminotransferase (ALT) > 40 U/L, aspartate aminotransferase (AST) > 40 U/L (upper limits of normal), or creatinine (Cr) > 73 μmol/L (upper limit of normal by enzymatic assay).
- Duplicate examination records—only the first record was retained.

### Sample size

As an exploratory analysis based on existing data, no prior sample size calculation was performed. A total of 64 cases and 541 controls were included, yielding a final sample size of 605 participants. Post-hoc power analysis was conducted to evaluate the detectability of the observed effects given the available sample size.

### Variable definitions

Outcome Variable: PUD status (case = 1, control = 0). Primary Exposure A: Corrected AG (AG_corr_, mmol/L), derived from venous serum biochemical measurements, serving as a reliable indicator of metabolic acid load. Primary Exposure B: HCO₃^−^ (mmol/L), obtained from venous serum biochemical testing. Adjustment Variables: Sex (male/female), age (years, continuous), and serum sodium (Na^+^, mmol/L) to account for potential confounding related to sex, age, and fluid volume status.

### Model definitions

To mitigate potential collinearity between AG_corr_ and HCO₃^−^—both physiological and statistical—and to elucidate their distinct pathophysiological roles, three complementary multivariable logistic regression models were constructed: (1) Model A (Metabolic Acid Load Model): Included AG_corr_ as the key exposure to specifically assess the independent association between metabolic acid load (reflecting accumulation of unmeasured anions) and PUD risk. (2) Model B (Alkaline Reserve Model): Included HCO₃^−^ as the key exposure to independently evaluate the effect of alkaline reserve status (reflecting the body’s buffering capacity against acid load) on PUD. (3) Model C (uncorrected AG Model): Included uncorrected AG as the key exposure. (4) Model D (ACAG Model): Included ACAG as the key exposure. All models were adjusted for sex, age, and serum sodium.

### A novel correction of the AG

The traditional formula for calculating AG, without considering ALB and other electrolytes, is: AG = [UA] − [UC] = [Na^+^] − ([Cl^−^] + [HCO₃^−^]). This formula has significant limitations as it disregards contributions from other measured cations (e.g., Ca^2+^, K^+^, Mg^2+^), anions (e.g., P), and ALB. Accordingly, we developed a comprehensive corrected AG formula that incorporates all the aforementioned indicators. Based on the principle of electrical neutrality: Total Positive Charge = Total Negative Charge. If “[]” represents the total charge contribution of the respective electrolyte, [Na^+^] + [K^+^] + [Ca^2+^] + [Mg^2+^] + [UC] = [Cl^−^] + [HCO₃^−^] + [ALB] + [P] + [UA]. Since [AG] = [Na^+^] − ([Cl^−^] + [HCO₃^−^]), therefore [AG] + [K^+^] + [Ca^2+^] + [Mg^2+^] + [UC] = [ALB] + [P] + [UA]; therefore [UA] − [UC] = [AG] + [K^+^] + [Ca^2+^] + [Mg^2+^] − [P] − [ALB], therefore AG_corr_ mmol/L = (AG + ΔK^+^ + ΔCa^2+^ + ΔMg^2+^ − ΔP − ΔALB) mmol/L, and ACAG = (AG − ΔALB) mmol/L. Among them, ΔALB = (measured ALB − normal ALB) × 0.25 (0.25 is the average charge correction factor for serum ALB (g/L), normal ALB ≈ 40 g/L), ΔK^+^ = (measured K − normal K) × 1 (1 is the charge coefficient for K^+^, normal K (mmol/L) ≈ 4 mmol/L), ΔCa^2+^ = (measured Ca − normal Ca) × 2 (2 is the charge coefficient for Ca^2+^, normal Ca (mmol/L) ≈ 2.4 mmol/L), ΔMg^2+^ = (measured Mg − normal Mg) × 2 (2 is the charge coefficient for Mg^2+^, normal Mg (mmol/L) ≈ 0.85 mmol/L), ΔP = (measured P − normal P) × 1.8 (1.8 is the average charge correction factor for serum phosphate ions, normal P (mmol/L) ≈ 1 mmol/L). Using the AG_corr_ instead of AG eliminates the falsely normal AG level caused by low ALB levels, thereby improving the detection of occult high-AG acidosis. AG_corr_ also isolates interference from abnormalities in K^+^, Mg^2+^, or P, more reliably reflecting the accumulation of metabolic acids (e.g., lactate, ketones).

At the physiological pH of human serum, inorganic phosphorus exists primarily as a mixture of HPO₄^2−^ and H₂PO₄^1−^. The ratio of the two is approximately 4:1 at the physiological pH of 7.4, as derived from the Henderson-Hasselbalch equation. Consequently, according to classical theory based on physicochemical principles, the weighted average charge correction factor is calculated as: (0.8 × 2) + (0.2 × 1) = 1.8. The ALB molecule, composed of 585 amino acids and rich in cysteine residues, adopts a negatively charged globular protein structure. Based on the modulation and refinement of the molecular charge of ALB, the charge-weighted correction factor was established, becoming an important parameter in hematological physiology research. The classical charge-weighted correction factor for ALB is 2.5. This coefficient is widely applied in hematology, pharmacology, and clinical therapeutics for the accurate assessment and rational utilization of ALB’s functions (41–44).

### Statistical analysis

A database was established using Excel software, and statistical analyzes were conducted mainly with SPSS version 26.0. All data points were retained in the analysis unless an unambiguous cause for abnormality was identified (e.g., batch effects, sample contamination, or instrument malfunctions). A *p* value less than 0.05 was deemed statistically significant. Categorical data were summarized using frequencies or composition ratios, whereas continuous data were represented by the mean ± standard deviation (*x̄* ± *s*) or median (interquartile range, IQR), as appropriate. A Chi-square test was performed for sex. Binary logistic regression was performed to identify influencing factors. Crude odds ratios (ORs) with 95% confidence intervals (CIs) were calculated to assess the univariate association between each independent variable and PUD. Collinearity was assessed using linear regression and quantified by the variance inflation factor (VIF). The sample size was estimated *a priori*, and the statistical power was evaluated *post hoc* using G-Power 3.1. Data were visualized using bar and box plots. A case–control matching was performed using R software (version 4.2.2) to match participants on sex and age, adjusting the case and control groups for sensitivity analysis.

## Results

### Descriptive statistics

**Figure 1:**
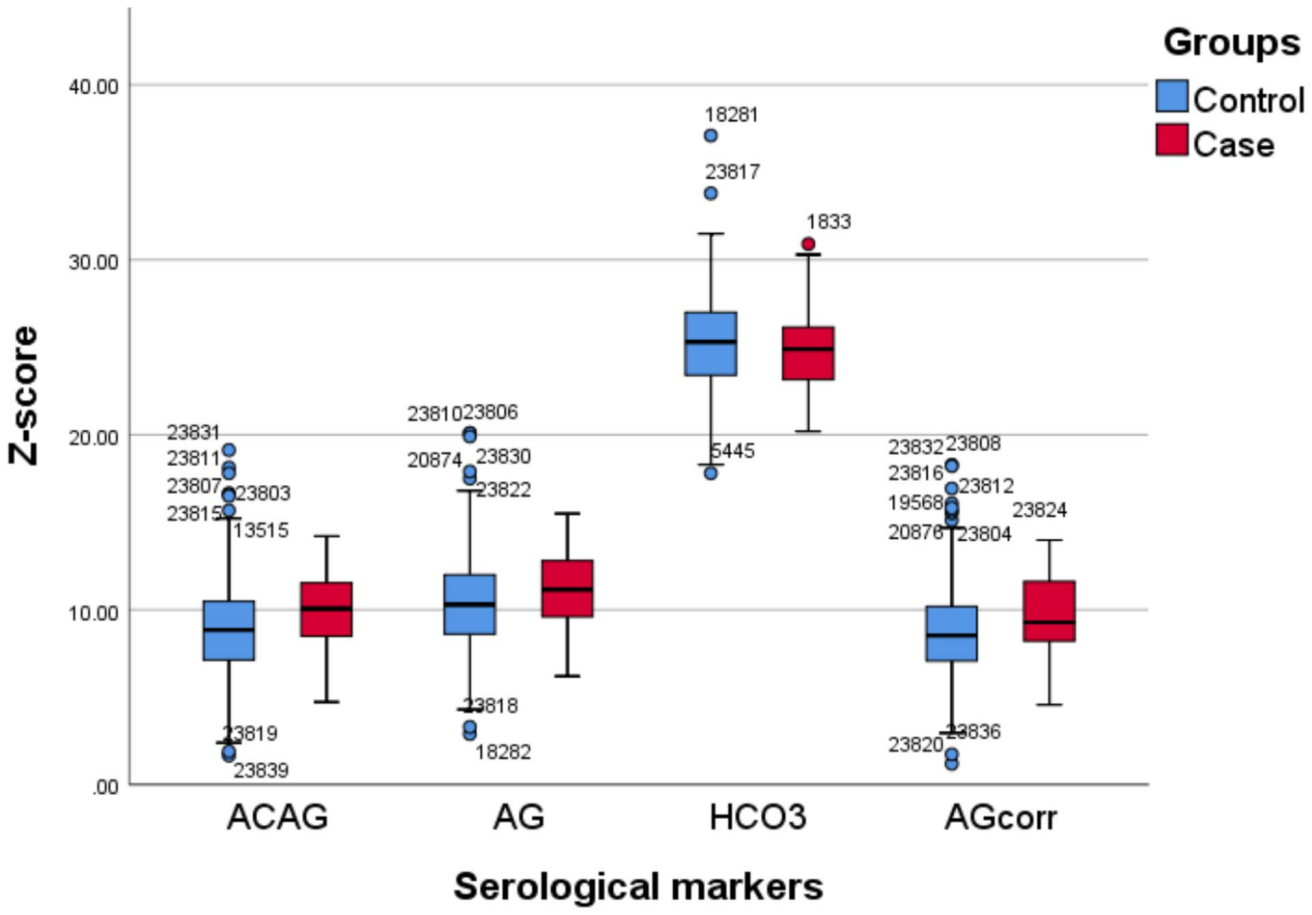
Boxplots of standardized values of HCO_3_^−^ and AG_corr_ in two groups.

The final analysis included 605 participants, comprising 64 patients with endoscopically confirmed PUD and 541 endoscopically confirmed non-PUD healthy controls, aged between 18 and 80 years. The control group consisted of 541 individuals (83 males, 458 females), while the case group included 64 patients (19 males, 45 females). Baseline characteristics of the study population are summarized in Table 1. The differences of age, sex, Na, HCO₃^−^, AG, AG_corr_, and ACAG between controls and cases was compared. Results showed significant differences in AG_corr_, ACAG, AG, Age and sex between the two groups (*p* < 0.05 or *p* < 0.001). There was no significant difference in Na and HCO₃^−^ between the two groups. See Figure 1 and Table 1 for details.

**Table 1:** Differences in indicators between two groups (*x̄* ± *s*) or [median (IQR)].

Variables	Overall data	Control group (*n* = 541)	Case group (*n* = 64)	*Z/χ* ^2^ */t*	*P*
Age (years)	45 (36, 54)	44 (35, 54)	52 (44, 58)	−4.354^a^	<0.001
Sex (male %)	16.86	15.34	29.69	8.402^b^	0.004
Na (*mmol/L*)	139.70 (138.30, 141.00)	139.60 (138.30, 140.95)	139.92 ± 2.07	−1.616^a^	0.106
AG_corr_ (*mmol/L*)	8.61 (7.20, 10.34)	8.53 (7.08, 10.20)	9.80 ± 2.21	−3.545^a^	<0.001
HCO₃^−^ (*mmol/L*)	25.13 ± 2.56	25.17 ± 2.59	24.78 ± 2.32	1.158^c^	0.247
AG (*mmol/L*)	10.50 ± 2.58	10.42 ± 2.61	11.15 ± 2.16	−2.146^c^	0.032
ACAG (*mmol/L*)	9.03 (7.40, 10.68)	8.85 (7.13, 10.48)	10.02 ± 2.19	−3.300^a^	0.001

“a” represents the *Z* value of the Mann–Whitney U test. “b” represents the χ^2^ value of the Pearson chi-square test. “*t*” represents the *t*-value of the *t*-test. AG, Anion gap; AG_corr,_ Corrected AG; ACAG, Albumin corrected AG.

### Univariate analysis

Univariate binary logistic regression analysis revealed significant associations of Sex, Age, AG_corr_, AG, and ACAG with PUD (*p* < 0.05), but not with HCO₃^−^ or Na. Details are provided in Table 2, and variable assignments are summarized in Table 3.

**Table 2:** Univariable binary logistic regression of factors associated with PUD.

Variables	*B*	SE	Wald *χ*^2^	*P*	OR	95%CI
Sex	0.846	0.298	8.030	0.005	2.330	1.298–4.182
Age	0.050	0.012	17.503	<0.001	1.051	1.027–1.076
Na	0.100	0.068	2.136	0.144	1.105	0.967–1.263
AG_corr_	0.170	0.052	10.899	0.001	1.185	1.072–1.311
HCO₃^−^	−0.060	0.052	1.341	0.247	0.942	0.851–1.042
AG	0.107	0.050	4.546	0.033	1.113	1.009–1.229
ACAG	0.147	0.050	8.462	0.004	1.158	1.049–1.278

**Table 3:** Variable assignment.

Category	Variable name	Assignment/values
Dependent variable	Group	0 = Control, 1 = PUD
Independent variables	Sex	0 = Female, 1 = Male
	Age	Original values
	Serological markers	

### Multivariate analysis

Multivariate binary logistic regression analysis was conducted in three models. Each model was distinguished by its key variable: Model A: AG_corr_; Model B: HCO₃^−^; Model C: uncorrected AG; Model D: ACAG. All models adjusted for sex, age, and sodium (Na). The results identified AG_corr_ (*B* = 0.155, *p* = 0.004) as a positive correlation factor for PUD. HCO₃^−^ (*B* = −0.112, *p* = 0.043) acted as a negative correlation factor. The uncorrected AG made a significant contribution (*B* = 0.104, *p* = 0.049), while the overall model showed a poor fit with the Hosmer-Lemeshow test (H-L test *χ^2^* = 17.895, *p* = 0.022). Model D was also significantly correlated (*p* < 0.05) but showed poor fit (H-L test *χ^2^* = 15.796, *p* = 0.045). Prior to performing multivariate logistic regression, collinearity diagnostics were conducted for all independent variables in the models. VIF analysis demonstrated that all variables in the models had values ranging from 1.0 to 1.2. All values were well below the threshold of 5, indicating the absence of severe multicollinearity in the models. Details are shown in Table 4; variable coding is provided in Table 3.

**Table 4:** Multivariate binary logistic regression for factors associated with PUD.

Models	Variable	*B*	SE	Wald *χ*^2^	*P*	OR	95%CI
Model A^a^	Sex	0.896	0.310	8.354	0.004	2.449	1.334–4.495
	Age	0.050	0.013	16.040	<0.001	1.051	1.026–1.078
	Na	−0.014	0.074	0.035	0.851	0.986	0.853–1.140
	AG_corr_	0.155	0.054	8.171	0.004	1.167	1.050–1.298
	Constant	−4.218	10.129	0.173	0.677	0.015	–
Model B^b^	Sex	0.960	0.309	9.651	0.002	2.612	1.425–4.788
	Age	0.053	0.013	17.936	<0.001	1.055	1.029–1.081
	Na	0.047	0.073	0.414	0.520	1.048	0.909–1.209
	HCO₃^−^	−0.112	0.055	4.113	0.043	0.894	0.802–0.996
	Constant	−8.633	9.915	0.758	0.384	0.000	–
Model C^c^	Sex	0.052	0.012	17.172	<0.001	1.053	1.028–1.079
	Age	0.913	0.308	8.785	0.003	2.492	1.362–4.558
	Na	−0.004	0.073	0.003	0.958	0.996	0.863–1.150
	AG	0.104	0.053	3.863	0.049	1.110	1.000–1.232
	Constant	−5.391	10.032	0.289	0.591	0.005	–
Model D^d^	Sex	0.934	0.309	9.117	0.003	2.544	1.388–4.664
	Age	0.050	0.012	16.283	<0.001	1.052	1.026–1.078
	Na	−0.009	0.073	0.016	0.900	0.991	0.858–1.144
	ACAG	0.138	0.053	6.856	0.009	1.148	1.035–1.273
	Constant	−4.773	10.098	0.223	0.636	0.008	–

“a” represents H-L test χ^2^ = 9.853, *p* = 0.276; Nagelkerke *R*^2^ = 0.115. “b” represents: H-L test 10.910, *p* = 0.207; Nagelkerke *R*^2^ = 0.102. “c” represents: H-L test χ^2^ = 17.895, *P* = 0.022; Nagelkerke *R*^2^ = 0.101. “d” represents: H-L test χ^2^ = 15.796, *P* = 0.045; Nagelkerke *R*^2^ = 0.111.

### *Post hoc* power analysis

#### Model A

Based on a total sample size of 605 and the observed effect (OR = 1.167), a *post hoc* power analysis indicated a statistical power of 89%. Sensitivity analysis revealed that the study conclusions were relatively sensitive to changes in sample size: when the sample size was reduced to 400, the power decreased to 75%, falling below the conventionally accepted threshold; when the sample size was increased to 800, the power rose to 95%. Considering the prior sensitivity analysis on effect size, this study possesses sufficient power to detect the observed effect but exhibits limited capability to withstand fluctuations in sample size or to detect weaker effects. See Table 5 for details.

**Table 5:** *Post hoc* power and sensitivity analysis results for models.

Models (target variable)	Scenario	OR	Power	*N*	Parameters
Model A (AG_corr_)	Baseline	1.167	0.89	605	*R*^2^ other *X* = 0.033, SD = 2.49
	Sensitivity (OR)				
	Conservative	1.05	0.23	605	Identical to baseline
	Mild conservative	1.10	0.55	605	Identical to baseline
	Optimistic	1.20	0.96	605	Identical to baseline
	Highly optimistic	1.30	0.99	605	Identical to baseline
	Sensitivity (N)				
	Insufficient N	1.167	0.75	400	Identical to baseline
	Sufficient N	1.167	0.95	800	Identical to baseline
Model B (HCO₃^−^)	Baseline	0.894	0.68	605	*R*^2^ other *X* = 0.058, SD = 2.56
	Sensitivity (OR)				
	Highly optimistic	0.805	0.99	605	Identical to baseline
	Optimistic	0.850	0.93	605	Identical to baseline
	Mild conservative	0.983	0.09	605	Identical to baseline
	Conservative	1.028	0.13	605	Identical to baseline
	Sensitivity (N)				
	Insufficient N	0.894	0.53	400	Identical to baseline
	Minimally sufficient	0.894	0.80	835	Identical to baseline
	Sufficient N	0.894	0.86	1,000	Identical to baseline
Model C (AG)	Baseline	1.110	0.64	605	*R*^2^ other *X* = 0.028, SD = 2.58
Model D (ACAG)	Baseline	1.148	0.84	605	*R*^2^ other *X* = 0.027, SD = 2.55

#### Model B

Although the association between HCO₃^−^ and the outcome reached statistical significance (OR = 0.894, 95% CI: 0.802–0.996, *p* < 0.05), a *post hoc* power analysis showed a baseline power of only 0.65. The sensitivity analysis revealed substantial uncertainty: if the true effect was near the lower confidence limit (OR = 0.805), the statistical power would be as high as 0.99; conversely, if the true effect was near the upper confidence limit (OR = 1.028), the power would be only 0.13. This indicates that the current estimate of the effect size is highly imprecise. Using G*Power analysis, with all other parameters held constant, approximately 835 participants would be required to reliably detect an effect size of OR = 0.894 with 80% power. See Table 5 for details.

#### Model C

The *post hoc* power analysis revealed that Model C had a power of only 0.64. Although the uncorrected AG showed a statistically significant association with PUD, its low post hoc power (0.64) and suboptimal model fit (H-L test *p* < 0.05) suggest that this finding lacks robustness and has limited statistical power. See Table 5 for details.

#### Model D

The post hoc power analysis revealed that Model D had a power of 0.84. ACAG showed a statistically significant association with PUD (*p* < 0.05), a post hoc power of 0.84, and suboptimal model fit (H-L test *p* < 0.05). It suggests that Model D is an “efficient but imprecise” detector. See Table 5 for details.

### Sensitivity analysis

#### Binary logistic regression analysis with adjusted control group

To assess the robustness of our findings, we performed a sensitivity analysis by altering the definition of the control group. We replaced the original controls (“Endoscopically confirmed, healthy individuals without PUD”) with a more broadly defined cohort (“Endoscopically confirmed, relatively healthy individuals without PUD”). The inclusion criteria for controls were expanded from the primary model’s requirement of a single diagnosis related to gastric mucosal status to allowing multiple benign endoscopic diagnoses, including conditions such as erosions, mucosal inflammation, and leukoplakia. The exclusion criteria remained consistent with the primary model.

The AG_corr_ level was 8.53 (7.08–10.20) in the primary model’s control group (*n* = 541) and 8.93 ± 2.52 in the sensitivity analysis’ adjusted control group (*n* = 1915). The case group (*n* = 64 active PUD patients) remained identical to the primary model.

The results demonstrated that the association between AG_corr_ and PUD remained statistically significant in the model with the adjusted control group (OR = 1.138, 95% CI: 1.029–1.259, *p* = 0.012). Although the inclusion of a more heterogeneous control group introduced potential confounders that could reduce statistical power, the post-hoc power of this model was 0.81, still exceeding conventionally accepted thresholds, and the model fit was good (H-L test *χ^2^* = 7.764, *p* = 0.457). This confirms that our core conclusion—metabolic acid load is a positive correlation factor for PUD—is robust to changes in the control group definition. See Table 6 for details.

**Table 6:** Sensitivity analysis for model A with an adjusted control group.

Model A	Control	AG_corr_ level (_mmol/L_)	*n* (case/control)	AG_corr_ OR (95% CI)	*P*	Power	H-L *P*
Primary	Healthy	8.53 (7.08, 10.20)	64/541	1.167 (1.050–1.298)	0.004	0.89	0.276
Sensitivity	Relatively healthy	8.93 ± 2.52	64/1915	1.138 (1.029–1.259)	0.012	0.81	0.457

#### Binary logistic regression analysis with case–control matching

Case–control matching was performed on sex and age to eliminate their effects on PUD. After matching, 289 cases were obtained, comprising 52 subjects in the case group (44 females, 8 males) and 237 subjects in the control group (219 females, 18 males). Binary logistic regression results indicated that sex, age, and Na did not have a significant effect on PUD (*B* = 0.150, *p* > 0.05), while AGcorr showed a significant effect on PUD (*p* < 0.05). Following the matching for sex and age, the explanatory power of the model was limited due to the elimination of a large number of control cases (Nagelkerke *R^2^* = 0.049). However, AGcorr still demonstrated stable significance, which is sufficient as a sensitivity analysis to support the robustness of the original model. See Table 7 for details.

**Table 7:** Sensitivity analysis with case–control matching on sex and age.

Variable	*B*	SE	Wald *χ^2^*	*P*	OR	95%CI
Sex	0.882	0.466	3.587	0.058	2.417	0.970–6.032
Age	0.009	0.017	0.304	0.581	1.009	0.976–1.044
Na	−0.045	0.089	0.259	0.611	0.956	0.804–1.137
AG_corr_	0.150	0.064	5.402	0.020	1.161	1.024–1.317
Constant	2.863	12.025	0.057	0.812	17.508	–

*n* (case) = 52, *n* (control) = 237. H-L test χ^2^ = 11.453, *p* = 0.177; Nagelkerke *R*^2^ = 0.049.

## Discussion

### Principal findings

First, this study developed a novel formula for calculating the AG. Second, this study employed two independent multivariable logistic regression models to investigate the association between acid–base balance markers—AG_corr_ and HCO₃^−^—and PUD in a population excluding individuals with abnormal liver or kidney function. On one hand, AG_corr_ demonstrated a robust and positive association with PUD; on the other hand, HCO₃^−^ suggested a potential negative association.

### Metabolic acid load and PUD

It is well established that gastric acid is the most critical aggressive factor in the pathogenesis of PUD. But is there a connection between gastric acid and systemic acid–base balance? In fact, existing studies have already revealed physiological mechanisms linking the two. It is possible that an important fact has long been overlooked: gastric acid secretion may serve as an important buffering pathway during episodes of metabolic acidosis in the body. Gastric acid secretion is primarily regulated by H^+^/K^+^-ATPase activity in gastric parietal cells (45, 46). The gastric H^+^, K^+^, -ATPase derives energy from the hydrolysis of ATP to transport H^+^ ions from the parietal cells of the gastric mucosa into the stomach in exchange for K^+^ ions. This is the mechanism of gastric acid secretion (47). Gastric acid secretion may be influenced by serum acid loading status. Electrolytes in the blood and across intracellular and extracellular compartments are exchanged and regulated through complex mechanisms (48). Excessive blood acid load may lead to increased gastric acid secretion, thereby exacerbating damage to the gastric mucosa. Studies have indicated that systemic acid–base imbalances modulate gastric acid secretion by influencing electrolyte transport and altering pH levels both intracellularly and extracellularly (49). Studies have shown that chronic metabolic acidosis in patients with chronic kidney disease (CKD) is associated with gastrointestinal mucosal damage and an increased risk of ulcers and bleeding (16, 17). In addition, a study of experimental acidotic mice revealed a significant increase in the transcript level of Atp4A and its protein expression in the stomachs of acidosis mice compared with control mice (50). The Atp4A gene encodes a key subunit of the proton pump responsible for gastric acid secretion. Taken together, the above discussion supports that an increased systemic acid load may elevate gastric acid secretion, which is the most significant aggressive factor in PUD. Therefore, there is reason to believe that the increase in metabolic acid load leads to PUD, rather than PUD causing the increase in metabolic acid load. Moreover, there is currently no evidence to suggest that peptic ulcer disease universally leads to an increased metabolic acid load.

The results of this study provide strong evidence from Model A that a statistically significant and reliable positive association exists between AG_corr_ and PUD (OR = 1.167, *p* < 0.05), supporting the inference that it acts as a risk factor for PUD. A chronic metabolic acidosis state may represent an important pathogenic mechanism of PUD. Post-hoc power analysis based on the observed effect size and current sample size (*N* = 605) indicated high statistical power (Power = 89%) to detect this association, supporting the robustness of the result. Sensitivity analysis of the OR suggested limitations in detecting weak effects; future research should focus on precisely quantifying the strength of this effect and further exploring its underlying mechanisms and practical implications. Sensitivity analysis with an adjusted control group showed that even under more stringent conditions—by including more potential confounders (which generally dilutes effect estimates)—the association between AG_corr_ and PUD remained statistically significant, with a statistical power of 0.81 for this comparison. Sensitivity analysis of case–control matching on sex and age also demonstrated the significant effect of AG_corr_ on PUD. This indicates that the effect of AG_corr_ on PUD is not coincidental and that the finding is robust. Such robustness substantially enhances the internal validity and scientific rigor of our conclusion and increases its potential relevance for clinical application.

From a pathophysiological perspective, elevated AG often suggests the accumulation of unmeasured anions (e.g., lactate, ketone bodies, uremic organic acids). After controlling for potential confounding from liver and kidney function, this study established that corrected AG can serve as a robust risk marker for PUD—i.e., the accumulation of metabolic acid load increases the risk of PUD. By excluding the influence of liver and kidney function, our findings imply that metabolic accumulation may represent a more direct mechanism in PUD pathogenesis. These results support the hypothesis that PUD may involve an underrecognized state of high metabolic acid load, and that this persistent metabolic stress could be an overlooked mechanism driving disease progression. In the context of PUD, AG_corr_ is more likely to be a proactive risk driver. Its robust association strongly suggests that the metabolic acid load represented by AG may itself be a previously overlooked pathological mechanism directly involved in the development of PUD. The acid-secreting function of the gastric mucosa may serve as an important pathway for systemic acid–base balance regulation (analogous to renal tubular acid secretion). A persistent systemic acid load may upregulate gastric acid secretion at the systemic level, thereby increasing aggressive factors acting on the gastric mucosa and contributing to the pathogenesis of PUD.

Therefore, the broader significance of this study lies in proposing a new perspective on PUD pathogenesis—an “metabolic acid attack” pathway that transcends the conventional intragastric microenvironment. We reveal that within the complex pathogenic network of PUD, in addition to local aggressive factors such as *H. pylori* and NSAIDs, a systemic metabolism-derived “metabolic acid attack” pathway may be equally important. Naturally, this “metabolic acid attack” hypothesis requires final confirmation through prospective studies, including direct comparisons and interaction analysis with traditional risk factors. Nonetheless, this study undoubtedly introduces a new metabolic dimension to understanding and addressing PUD—a classic condition.

### Methodological innovation: the value of the fully-corrected AG formula in PUD assessment

Another major advance of this study lies in its methodological breakthrough. Building upon the simplified formula AG = [Na^+^] – ([Cl^−^] + [HCO₃^−^]), we have developed and validated a comprehensive AG correction formula. Unlike traditional approaches that adjust only for ALB or focus on individual electrolytes, our formula systematically integrates potassium (K^+^), magnesium (Mg^2+^), calcium (Ca^2+^), phosphorus (P), and ALB into the AG calculation and has applied it in our models. The inclusion of these parameters is grounded in solid physiological and biochemical rationale. The traditional AG ignores the influence of these factors, potentially leading to significant discrepancies between the estimated AG and the true metabolic acid load. Consequently, our new model aims to integrate these key parameters to construct an assessment tool that more accurately approximates the “true” metabolic acid load *in vivo*. The results of this study provide clinically useful reference values: the AG_corr_ levels were 8.53 (7.08–10.20) in the Model A control group (541 adults) and 8.93 ± 2.52 in the adjusted control group (1915 adults) of the sensitivity analysis model.

The comparison between Model A and Model C underscores the necessity of AG correction. The results from Model C (uncorrected AG) provide crucial counter-evidence highlighting the importance of comprehensive correction. Although the uncorrected AG also showed statistical significance, its poor model fit (H-L test *p* < 0.05) and low statistical power (0.64) collectively indicate that the uncorrected AG is a crude and unreliable risk indicator. The disadvantages of Model C, when contrasted with the robust and efficient Model A, highlight the exceptional value of the fully-corrected AG—representing a transition from crude to precise, and from inefficient to efficient. This comparison strongly demonstrates that comprehensive AG correction is not an optional refinement but a critical step in transforming AG from an unstable signal into a robust biomarker. It removes the “noise” introduced by variations in other electrolytes and proteins, allowing us to capture the true effect of the metabolic acid attack on PUD more clearly and reliably.

The results from Model D (ACAG Model) indicate that eliminating ALB interference is a necessary step to unlock AG’s predictive potential, resulting in a leap in its statistical power. However, its suboptimal model fit also exposes limitations, suggesting that while ALB correction is necessary, it is not sufficient. The final Model A demonstrates that only through comprehensive correction, which simultaneously accounts for electrolytes like potassium, magnesium, calcium, and phosphorus that collectively form the acid–base balance network, can an ideal biomarker be obtained—one that is both sensitive and well-calibrated.

There is currently no universally accepted formula for AG correction, and numerous studies have tailored their correction approaches to their specific research objectives, achieving robust results (27–30). This study developed a novel fully-corrected AG formula suitable for assessing metabolic acid load (as opposed to alkali loss) and demonstrated its superiority over traditional AG and ACAG in PUD-related mechanistic investigations, showing robust sensitivity. Our findings confirm that the fully-corrected AG index demonstrates robust sensitivity in detecting disturbances associated with PUD. This discovery strongly suggests a possible widespread prevalence among PUD patients of a subtle yet persistent “subclinical metabolic acidosis” state caused by a “high metabolic acid load”—a condition previously masked by the insufficient sensitivity of detection tools. The nature of this state may stem from a chronic imbalance between metabolic acid production and clearance, potentially due to complex dietary patterns or specific metabolic phenotypes. In terms of clinical translation, this work theoretically opens new perspectives for understanding the systemic metabolic etiology of PUD, revealing an metabolic acid attack pathway independent of traditional local factors. In the future, this fully-corrected AG formula holds promise as a sensitive biological marker to be integrated into PUD risk prediction models.

### HCO₃^−^ and PUD

Based on physiological principles, the body’s alkaline reserve can counteract acidic metabolites. Therefore, if metabolic acid load is a risk factor for PUD, then the alkaline buffer system that counteracts it would be a protective factor. Our findings suggest a potential negative correlation between HCO₃^−^ and PUD, supporting the inference that it acts as a protective factor. For Model B (the HCO₃^−^ model), the current study had only 65% power to detect the observed effect (OR = 0.894). The present sample size is insufficient to precisely estimate an effect close to the null boundary. The low baseline power (0.65) and extreme sensitivity analysis results indicate that we cannot reliably distinguish whether HCO₃^−^ has a substantial protective effect (OR = 0.802) or is nearly ineffective (OR = 0.996). Although the association was statistically significant, this finding may lack robustness, and the true effect is likely weaker than the point estimate suggests. Sample size sensitivity analysis provides clear guidance: the current sample size of 605 is clearly inadequate for verifying the HCO₃^−^ effect (power = 0.65). It would need to be increased to 835 subjects to reach the acceptable power threshold (0.80), or to 1,000 subjects to achieve robust statistical power (0.86).

### Alignment with and extension of existing theoretical consensus

Traditionally, PUD and stress ulcers (SU) have been regarded as having distinct pathogenic mechanisms, and their etiologies have rarely been linked. However, this study may challenge this conventional separation, as an elevated systemic acid load could represent a shared underlying mechanism for both conditions. A substantial amount of evidence from previous literature readily supports the association between acidosis and SU. Jiang Lanting et al. reported that SU are associated with elevated lactic acid levels (51), while Li Bing et al. found that metabolic acidosis is one of the independent risk factors for severe craniocerebral injury complicated by SU (52). Kivilaakso suggested that metabolic acidosis characterized by “low HCO₃^−^” can reduce HCO₃^−^ supply to the mucosa, thereby increasing its susceptibility to ulcers (53). Other scholars have reported that metabolic acidosis is a significant risk factor for acute spinal cord injury and SU (54). A patient with SU induced by thyrotoxicosis was complicated by high anion-gap acidosis (55). Furthermore, multiple studies globally have documented that patients with diabetic ketoacidosis (DKA) frequently develop SU or experience gastrointestinal bleeding. Hao et al. (56), Uhlenhopp et al. (57), Badipatla et al. (58) propose that lactate levels can be used as a predictive marker for gastrointestinal bleeding (58). Therefore, we can speculate that acidosis, particularly metabolic lactic acidosis, may be a fundamental mechanism underlying the development of SU. The correlation between acute stress ulcers and metabolic acid load may underpin the pathogenesis of PUD.

### Strengths and limitations

The primary strength of this study lies in our proactive exclusion of individuals with abnormal liver or kidney function, which effectively controlled for these key confounding factors. Additionally, by adjusting for the influences of sex, age, and fluid volume differences, we were able to more clearly reveal the relationship between PUD itself and acid–base balance. Furthermore, moving beyond mere reporting of *p*-values, we employed post-hoc power and sensitivity analysis to provide quantitative support for the robustness of our conclusions, adhering to a more transparent and responsible reporting standard.

This study has several limitations. Binary logistic regression results from Model A indicated that both sex and age had significant effects on PUD. To eliminate their influence on PUD, we performed case–control matching for sex and age. The subsequent binary logistic regression analysis showed that AG_corr_ remained a significant factor for PUD, confirming that its effect is independent of sex and age. Due to the inherent nature of the retrospective design, we were unable to systematically obtain and adjust for all participants’ *H. pylori* infection status and NSAIDs use, both of which are well-established risk factors for PUD. This may lead to residual confounding and represents an important consideration when interpreting our results. In addition, regarding the analysis of HCO₃^−^, it is possible that its effect is inherently extremely weak and unstable, or that our sample size was insufficient. Although this study found that it has a potential protective effect on PUD, further research with a larger sample size is needed to clarify its exact role.

## Summary

After adjusting for sex, age, and Na, AG_corr_ remained an independent risk factor for PUD in individuals with normal liver and kidney function. Metabolic non-volatile acid attack may be an important pathogenic mechanism in PUD. Through our original comprehensive correction formula, we transformed AG into a highly sensitive indicator, revealing for the first time a prevalent “subclinical metabolic acidosis” state among PUD patients—a finding that warrants substantial attention from clinicians. The fully-corrected AG demonstrated significantly superior model fit and statistical power compared to the uncorrected AG and ACAG, establishing it as a robust and reliable biomarker. We recommend its integration into clinical PUD risk assessment and disease management pathways to facilitate a shift toward earlier and more proactive prevention and treatment strategies. Furthermore, we also discovered that the HCO₃^−^ might be a potential protective factor, but its clinical significance still needs to be further confirmed. From a nutritional perspective, correcting the acid–base imbalance caused by non-volatile acids and reducing AG_corr_ could be a key mitigating strategy for preventing and treating PUD. Increasing the alkaline reserve may also be a potential therapeutic approach. These findings require further validation in broader populations to better control for confounding factors and enhance their generalizability.

## Data Availability

The original contributions presented in the study are included in the article/supplementary material, further inquiries can be directed to the corresponding author.
